# Overcrowded housing increases risk for COVID-19 mortality: an ecological study

**DOI:** 10.1186/s13104-022-06015-1

**Published:** 2022-04-05

**Authors:** Karan Varshney, Talia Glodjo, Jenna Adalbert

**Affiliations:** 1 Deakin University School of Medicine, 75 Pigdons Road, Waurn Ponds, VIC 3216 Australia; 2 Jefferson College of Population Health, Philadelphia, PA Australia; 3 Sidney Kimmel Medical College at Thomas Jefferson University, Philadelphia, PA Australia

**Keywords:** COVID-19, Mortality, Housing, Inequities, Prevention

## Abstract

**Objectives:**

Overcrowded housing is a sociodemographic variable associated with increased infection and mortality rates from communicable diseases. It is not well understood if this association exists for COVID-19. Our objective was hence to determine the association between household overcrowding and risk of mortality from COVID-19, and this was done by performing bivariable and multivariable analyses using COVID-19 data from cities in Los Angeles County.

**Results:**

Bivariate regression revealed that overcrowded households were positively associated with COVID-19 deaths (standardized β = 0.863, p < 0.001). COVID-19 case totals, people aged 60+, and the number of overcrowded households met conditions for inclusion in the backwards stepwise linear regression model. Analysis revealed all independent variables were positively associated with mortality rates, primarily for individuals 60 + (standardized β_1_ = 0.375, p = 0.001), followed by overcrowded households (standardized β_2_ = 0.346, p = 0.014), and total COVID-19 cases (standardized β_3_ = 0.311, p < 0.001). Our findings highlight that residing in overcrowded households may be an important risk factor for COVID-19 mortality. Public health entities should consider this population when allocating resources for prevention and control of COVID-19 mortality and future disease outbreaks.

## Introduction

As of November 1, 2021, over 750,000 deaths in the United States (US) have been attributed to COVID-19 infection [1]. In the US, mortality among individuals with communicable and non-communicable disease is disproportionately higher for those with poor socioeconomic circumstances [2, 3]. Household size, defined as the number of individuals occupying one household, is a key sociodemographic variable related to the spread of disease. “Household overcrowding” is a term applied to households in which the number of occupants surpasses the number of rooms, and is disproportionately prevalent among Hispanic persons, persons living in rented homes, persons not born in the US, households earning less than $25,000 per year, in the Western US, and urban areas [5]. In particular, household overcrowding has been historically associated with an increased incidence in infectious pathogens, such as helminths and tuberculosis [4].

Although larger household size and overcrowding have been associated with a greater incidence of COVID-19 infection, there has been limited research conducted on the effect that household overcrowding has on COVID-19 mortality rates [6, 7]. Critically, however, one study has demonstrated a possible link between COVID-19 mortality and total number of overcrowded households, though the evidence was limited and hence the authors suggested a need to study this association in more detail [8]. Given the paucity of research in this area and its potential impact on future research and data acquisition in a pandemic setting, the purpose of this ecological study was to analyze the association between household overcrowding and mortality from COVID-19.

## Main text

### Methods

Los Angeles (LA) County has the greatest population density in the US [9] and has recorded the highest number of COVID-19 cases (> 1,240,000) and deaths (> 24,000) in the nation [10]. COVID-19 data was therefore acquired for all cities in LA County [11], along with data on housing and demographics up until July 28, 2021 [9]. Institutional Review Board approval was not required, as all data used for this study is publicly available.

Overcrowded households were defined as having 1.0 + persons per room. Bivariate regression was performed between the number of overcrowded households and the number of COVID-19 deaths. Backwards stepwise linear regression was then conducted with risk factors for COVID-19 mortality, such as race, sex, level of income, and age as eligible input variables. Collinearity was assessed by considering the variance inflation factors (VIF); variables with high collinearity (VIF > 8) were removed from the model.

### Results

Data was fully available for 85 of the 88 cities in LA County. Of these 85 cities, there were a total of 540,155 COVID-19 cases, 10,947 COVID-19 deaths, and 6,784 overcrowded households. Full descriptive statistics of variables considered for analysis are listed in Table 1.

**Table 1:** Descriptive statistics for cities of LA County

Variable	Total across 85 cities	Median (range)
COVID-19 cases	540,155	991 (19–25,582)
COVID-19 deaths	10,947	93 (0–633)
Overcrowded households	138,755	987 (0–6784)
Males	2,193,265	19,212 (42–103,918)
0–19 years of age	1,146,966	9722 (23–59,833)
20–59 years of age	2,467,903	22,311 (49–114,242)
60 + years of age	862,657	8316 (18–47,832)
Black race	306,691	840 (0–46,326)
Hispanic race	2,106,564	14,613 (60–109,103)
Median household income	7,159,521	71,948 (39,738–239,375)
Unemployed (above 16 years of age)	147,380	1302 (2–7566)

Bivariate regression indicated that the number of overcrowded households was positively associated with the number of COVID-19 deaths (standardized β = 0.863, p < 0.001). A stronger association was seen between COVID-19 cases and deaths (standardized β = 0.892, p < 0.001).

Of the eligible variables, three met the conditions for inclusion in the backwards stepwise linear regression model: total COVID-19 cases, the number of individuals aged 60 + , and total overcrowded households. The analysis revealed that all three of these independent variables were positively associated with the number of COVID-19 deaths. The largest effect was seen in individuals aged 60+ (standardized β_1_ = 0.375, p = 0.001), followed by overcrowded households (standardized β_2_ = 0.346, p = 0.014), and total COVID-19 cases (standardized β_3_ = 0.311, p < 0.001). For each of the three variables, results of the analyses are listed in Table 2.

**Table 2:** Association with COVID-19 mortality* for bivariate and multivariable analysis of eligible variables

	Bivariate analysis			Multivariable analysis		
	Unstandardized β (95% CI)	Standardized β	p-value	Unstandardized β (95% CI)	Standardized β	p-value
Overcrowded households	0.063 (0.54, 0.071)	0.863	p < 0.001	0.025 (0.013, 0.037)	0.346	< 0.001
COVID-19 cases*	0.017 (0.015, 0.019)	0.892	p < 0.001	0.006 (0.003, 0.009)	0.311	0.001
Individuals age 60 +	0.012 (0.010, 0.014)	0.825	p < 0.001	0.005 (0.004, 0.007)	0.375	< 0.001

*COVID-19 case and death data from as of July 28, 2021

### Discussion

Per the results of our analysis, household overcrowding is a significant risk factor for COVID-19 mortality. Importantly, the results of our study revealed that in LA County, household overcrowding was an even stronger predictor of increased mortality rates than the total number of COVID-19 cases. Additionally, our findings emphasize that elderly citizens residing in overcrowded households are at a particularly elevated risk of mortality from COVID-19.

These findings suggest key implications for addressing the COVID-19 pandemic and future outbreaks of communicable disease. These findings are consistent with studies investigating COVID-19 transmissibility which found transmission to be greater in indoor congregate settings, such as jails and buses [12]. These settings share similar characteristics with overcrowded housing, including prolonged time spent with the same group of individuals, minimal ventilation, and multiple individuals occupying a limited space. While age, level of income, ethnic background, and medical co-morbidities have been frequently described as risk factors for poor outcomes associated with COVID-19 infection [13], our analyses suggest that public health measures designed to reduce mortality among persons with COVID-19 ought to make special consideration for persons living in overcrowded housing.

The Centers for Disease Control and Prevention (CDC) has suggested that infected individuals maintain six-foot distance between themselves and other household members to reduce transmission through the air by droplets and aerosols [14]. However for persons living in overcrowded housing, complying with this recommendation may be difficult or impossible. Developing recommendations that aim to specifically address the unique needs of persons living in overcrowded housing may improve the health outcomes for this group. For example, it has been previously recommended for emergency accommodations to be offered for those with unstable/unsafe housing after contraction of COVID-19 [15]. In addition, public health entities and healthcare providers should assess the prevalence of household overcrowding in the populations that they serve to inform interventions and more effectively allocate resources for COVID-19 prevention and control.

More broadly, this study underlines how this pandemic has exacerbated the detrimental effects of the housing crisis in the US on the health of the population, and the urgent need to increase access to affordable housing to reduce morbidity and mortality from COVID-19. Alongside the need to increase affordable housing, additional efforts to support those residing in overcrowded households can have a positive impact on health outcomes. Examples of such efforts may include public health agencies working with communities to improve current household conditions [16], increasing availability of social housing [17], and offering rent support to assist individuals in moving out of overcrowded households [18].

Household overcrowding may increase the risk of COVID-19 mortality. Public health agencies should recognize the importance of effectively allocating resources to areas with overcrowded housing during the COVID-19 pandemic and future disease outbreaks. Our findings emphasize an imperative for further studies to explore the association between overcrowded housing and COVID-19 mortality, as well as mortality attributed to other communicable pathogens.

## Limitations

Limitations of our work include that our ecological analysis can only provide partial insights regarding the additional barriers experienced by populations in overcrowded housing, such as discrimination or social exclusion. Furthermore, we were unable to account for undocumented or homeless individuals, which are equally important populations to consider when addressing infection and mortality rates. In addition, the health status of individuals within households was not able to be accounted for; it is possible that the number of individuals with a poor health status, or a high number of comorbidities, may have served as confounding variables. Finally, while the cities in LA County encompass a large portion of the County’s population, they do not account for unincorporated areas (regions not governed by municipal corporations), which comprise a sizeable proportion of the County. Regardless of these limitations, our study emphasizes the imperative for further research and data acquisition on the association between household overcrowding and mortality due to COVID-19 infection.

## Data Availability

The datasets used and/or analyzed during the current study are available from the corresponding author on reasonable request.

## Abbreviations

US: United States
LA: Los Angeles
VIF: Variance inflation factors
CDC: Centers for Disease Control and Prevention
